# Spatiotemporal stability of DUS characteristics in maize and evaluation of spatial replication testing models

**DOI:** 10.3389/fpls.2026.1854476

**Published:** 2026-09-28

**Authors:** Xiongfei Jiao, Jin Yu, Xiaofan Shi, Yufeng Wang, Jiaying Li, Leyong Feng, Zhi Zhang, Ruixi Han, Han Zhang

**Affiliations:** 1 Maize Research Institute, Shanxi Agricultural University, Xinzhou, China; 2 Shanxi Agricultural University, High Latitude Crops Instituteto Shanxi Academy, Datong, China; 3 Division of New Plant Variety Testing, Development Center of Science and Technology, Beijing, China; 4 Crop Research Institute, Shandong Academy of Agricultural Sciences, Jinan, China

**Keywords:** core characteristic, DUS testing, maize, spatial replication mode, spatio-temporal stability

## Abstract

Distinctness, uniformity, and stability (DUS) testing is the statutory basis and core element for the protection and registration of new plant varieties. In this study, 116 genetically diverse maize accessions (inbred lines, CS hybrids, CH) were evaluated using phenotypic data from Xinzhou and Datong (2024–2025). The spatiotemporal stability of 39 DUS characteristics was systematically analyzed using coefficient of variation (CV), principal component analysis (PCA), and UPGMA clustering. The results showed the following: (1) characteristics were categorized into three stability groups based on CV quantitative analysis, with 20 characteristics identified as spatiotemporally stable to serve as a routine observation baseline, while C2, C25, and C30.2 were recognized as time-sensitive characteristic. (2) PCA indicated that the spatial environment primarily controlled phenotypic variation (cumulative variance contribution: 74.92%). An optimized evaluation system using seven core characteristics, including plant height and grain type, was established to simplify the DUS testing process. (3) Validation showed that the spatial repetition model achieved a cluster retrieval (CR) rate of 92.31%, with accuracies of 94.36% for distinctness and 96.23% for uniformity. These values significantly exceeded those of the traditional temporal model, effectively reducing the impact of environmental noise and annual climatic variation. This study established an efficient spatial repetition testing paradigm based on characteristic stability and demonstrated the scientific feasibility of reasonably shortening testing cycles, thereby providing a rigorous scientific foundation for optimizing Chinese maize DUS testing standards and enhancing the efficiency of substantive examinations.

## 1 Introduction

Maize (*Zea mays* L.), as a primary crop for food, feed, and industrial raw materials in China, occupies a pivotal strategic position within the national food security guarantee system (Zhang et al., 2023; Kassem et al., 2024). According to the Seed Law of the People’s Republic of China and the regulations for the protection of new plant varieties, a variety must satisfy the criteria of distinctness, uniformity, and stability (DUS). DUS testing provides the legal technical basis for the substantive examination, release, and registration of plant variety rights (Chen et al., 2024; Zhu et al., 2024). At present, maize DUS testing in China is strictly performed following the national standard “Guidelines for the Conduct of Tests for Distinctness, Uniformity and Stability: Maize” (GB/T 19557.24—2018) (hereafter the “Guidelines”) (Yu et al., 2024). As required by the Guidelines, since the expression of quantitative characteristics (QN) in maize is highly influenced by environmental conditions, field trials over at least two independent growing cycles are generally necessary, with example varieties included to correct for environmentally induced variation. However, with the enhancement of China’s seed industry innovation capacity, the volume of new variety applications has surged annually, and the prolonged testing cycles and high costs associated with the current model have become bottlenecks restricting the market transformation of elite varieties and the efficiency of seed industry revitalization (Yang et al., 2023).

Internationally, a short-term testing model for *Phaseolus vulgaris* (Pramanik et al., 2023) has been developed in the Netherlands, and France (Zhang et al., 2022) has pioneered a “one year, two locations” spatial replication protocol for DUS testing in maize inbred lines (CS), which overcomes the constraints of single-site observations via multi-location data and reduces testing duration without compromising precision. In China, although research on shortening the DUS testing cycle has explored areas such as greenhouse generation acceleration (Fu and Wang, 2023; Navreet Kaur et al., 2024), correlation between multi-location sites, and phenotypic plasticity evaluation, a mature, reliable, and scalable standardized technical system that complements traditional models has yet to be established (Muthoni and Nyamongo, 2008; Jamnadass et al., 2020; Yang et al., 2021). Based on this, systematically analyzing the spatiotemporal stability characteristics of basic DUS characteristic and clarifying the variation patterns of different environment-sensitive characteristics is of great significance for constructing an efficient evaluation system based on characteristic stability and driving the transition of DUS testing toward precision design.

China is a major global power in maize production, consumption, and trade. According to data released by the National Bureau of Statistics of China, the maize planting area in China reached approximately 43 million hectares in 2025, ranking first globally, while the production is estimated at 301.2 million tons, accounting for 23% of world output and ranking second (Jin et al., 2023). At the same time, maize is grown across diverse regions in China, where environmental heterogeneity significantly influences the expression of phenotypic characteristics (Shu et al., 2023). Xinzhou (Su et al., 2023) and Datong (Zhao et al., 2014) in Shanxi Province are major spring maize production areas (Zhang et al., 2010) in North China, situated in the very early maturing spring maize region (Li et al., 2024). These areas offer representative yet specific ecological conditions and, as national DUS testing centers, provide the necessary data and facilities for studying the spatiotemporal stability of phenotypic characteristics.

Given this background, this study investigates phenotypic characteristics involved in maize DUS testing using data collected in 2024 and 2025 at Xinzhou and Datong. Multivariate statistical techniques, including coefficient of variation (CV), principal component analysis (PCA), and cluster analysis, were employed to evaluate site suitability and identify core characteristics for DUS testing. The specific research logic is as follows: first, quantify the variation magnitude of different phenotypic characteristics across spatiotemporal dimensions via CV analysis to define the classification features of spatiotemporally stable, spatially sensitive, temporally sensitive, and dual spatiotemporally sensitive characteristics. Second, perform dimensionality reduction on phenotypic characteristic through PCA to eliminate redundant characteristic and screen for core evaluation indicators. Finally, utilize cluster resonance (CR) quantitative analysis to verify the stability of maize variety classification across different spatiotemporal sites and clarify the application efficacy and feasibility of the spatial repetition testing model in maize DUS testing. This research aims to clarify the spatiotemporal stability patterns of maize DUS testing characteristic and construct an efficient, accurate, and low-cost technical model for maize DUS testing, providing scientific support for optimizing Chinese maize DUS testing standards and improving testing efficiency and quality. Furthermore, the findings can provide important theoretical references for maize genetic breeding, regional adaptability evaluation, and market-oriented promotion, carrying significant theoretical and practical value for promoting the high-quality development of China’s maize seed industry and safeguarding national food security.

## 2 Materials and methods

### 2.1 Experimental materials

A total of 116 maize germplasm resources with broad genetic representativeness, comprising 20 inbred lines and 96 hybrids (**Supplementary Table S1**), were selected from Xinzhou in 2024 and 2025 and from Datong in 2025, all of which served as applicant varieties for Plant Variety Protection (PVP) or DUS testing. Among these, 97 applicant varieties were without close varieties, while nine applicant varieties were paired with 10 corresponding close varieties. A total of 116 maize accessions were sourced from commercially released cultivars, regional trial lines, and intermediate breeding germplasm originating from Shanxi Province and its neighboring provinces over the preceding 3 years. This panel spans three maturity classes (early, medium, and late maturity), two germplasm categories (single-cross hybrids and inbred lines), and four endosperm phenotypes (normal, waxy, sweet, and sweet-waxy). Random sampling was performed to ensure representative genetic diversity across the test panel. Candidate similar varieties were primarily pre-screened according to phenotypic similarity assessed from field observations based on historical phenotypic data acquired in the preceding cropping season or published literature. Subsequent validation was supplemented by SSR genotyping using a core set of 40 primer pairs, with a threshold of ≥85% genetic similarity for final confirmation (GB/T 39914-2021). Although subjective bias cannot be entirely eliminated during evaluation, all phenotypic assessments were independently conducted by two separate evaluators.

### 2.2 Experimental design

#### 2.2.1 Test site

##### 2.2.1.1 Time span and field design

The overall experimental period was set from 2023 to 2025, with the core analysis focusing on observation data from two complete growing cycles in 2024 and 2025 to ensure the reliability and representativeness of the findings. Different field layouts were used: For test groups comprising ≤7 candidate similar varieties, the adjacent paired planting arrangement with two intra-block replications was applied; groups with more than seven entries were arranged under a randomized complete block design (RCBD) featuring three replications to reduce experimental error variance. For historical varieties with pre-existing test reports, an adjacent planting method was uniformly adopted to maintain data continuity and comparability. To achieve standardized calibration of phenotypic characteristic in DUS testing, sets of example varieties were interplanted at regular intervals to adjust for the impact of environmental heterogeneity on characteristic observations. Simultaneously, four protection rows were established around the experimental field to effectively attenuate edge effects and external environmental interference, ensuring a uniform growth environment for all tested varieties.

##### 2.2.1.2 Cultivation management and data quality control

Standard agronomic practices were followed at all sites, with sowing dates specifically adjusted to April 26 in Xinzhou and May 15 in Datong according to local environmental conditions (**Table 1**). This design not only adheres to the standardization principles of DUS testing, ensuring the consistency and controllability of field management measures, but also accurately matches the actual production conditions of different ecological zones. It authentically restores the phenotypic expression characteristics of varieties under natural growth environments, providing a reliable experimental foundation for systematically parsing the regulatory effects of environmental factors on DUS characteristic expression. For characteristic observation, a dedicated “same crop, same tester” responsibility system was strictly enforced. Professionally trained and qualified permanent personnel were responsible for all DUS characteristic evaluations of all tested varieties across both sites throughout the year, fundamentally eliminating differences in judgment scales between various testers and ensuring the consistency and comparability of observation data from different locations. Simultaneously, stringent quality control standards were formulated: critical, time-sensitive characteristics such as anthesis and maturity stages had to be observed across the entire plot within a 48-h window to minimize phenotypic errors arising from temporal spans. During the observation process, example varieties served as reference benchmarks for calibration. Should a deviation in characteristic data exceed 5%, the entire observation protocol was immediately re-initiated. Original field record sheets were uniformly scanned and archived, and data organization was conducted using an Excel double-blind entry method to ensure a recording error rate below 0.1%, providing high-precision data support for subsequent cluster analysis and stability assessment.

**Table 1:** Environmental description of the experimental site.

Environment	Sowing date	Harvest date	Latitude	Longitude	Average altitude (m)	Annual average temperature (°C)	Annual average precipitation (mm)	Annual sunshine hours (h)	Soil property	pH	Previous crop
Env-1 2025 Plant New Variety Test (Xinzhou) Sub-center, MARA	2025/4/26	2025/9/24	38°42′ N	112°73′ E	800 m	10.1	442	2,305	Loam	7.2–8.5	Maize
Env-2 2025 SAAS Experimental Base, Maozao, Datong	2025/5/15	2025/10/8	39°92′ N	113°27′ E	1,040 m	6.7	408	2,765	Sandy loam	7.9–8.6	Foxtail millet
Env-3 2024 Plant New Variety Test (Xinzhou) Sub-center, MARA	2025/4/30	2025/10/1	38°42′ N	112°73′ E	800 m	10.0	445	2,290	Loam	7.2–8.5	Maize

### 2.3 Characteristic investigation and data processing

#### 2.3.1 Investigation standards and characteristic list

Phenotypic data collection for all accessions followed the International Union for the Protection of New Varieties of Plants (UPOV) maize testing guidelines (TG/2/7) and the Chinese national standard (GB/T 19557.24—2018) for DUS testing in maize. A total of 39 characteristics were evaluated, of which 12 were quantitative (measured) characteristics (**Table 2**). Quantitative characteristics were measured at specific biological stages of plant maturity using standardized instruments and uniform operational procedures to maximally reflect their genetic potential and maintain high data comparability. The assessment of qualitative and pseudo-qualitative characteristics was performed in strict accordance with the standardized grading scales provided in the UPOV guidelines for characterization and documentation. All characteristic investigations were executed by professional technical personnel who had received systematic training. Within the measured characteristic, distinct codes of “1” and “2” were utilized to differentiate between inbred lines and hybrids, respectively, for the same characteristic.

**Table 2:** Characteristics for DUS testing.

Characteristic code	Characteristic type	Characteristic type	Observation method	Characteristic category
C1	First leaf: shape of apex	PQ	VG	Group visual characteristic, type I
C2	First leaf: anthocyanin coloration of sheath	QN	VG	Group visual characteristic, type I
C3	Foliage: intensity of green color	QN	VG	Group visual characteristic, type I
C8	Leaf: anthocyanin coloration of blade margin	QL	VG	Group visual characteristic, type I
C9	Ear: anthocyanin coloration of silks	QN	VG	Group visual characteristic, type I
C10	Tassel: anthocyanin coloration of anthers	QN	VG	Group visual characteristic, type I
C12	Tassel: anthocyanin coloration at base of glume	QN	VG	Group visual characteristic, type I
C13	Tassel: anthocyanin coloration at tip of glume	QN	VG	Group visual characteristic, type I
C14	Tassel: anthocyanin coloration of glumes excluding base	QN	VG	Group visual characteristic, type I
C22	Stem: anthocyanin coloration of brace roots	QN	VG	Group visual characteristic, type I
C25	Leaf: anthocyanin coloration of sheath	QN	VG	Group visual characteristic, type I
C33	Ear: shape	QN	VG	Group visual characteristic, type I
C34	Ear: number of colors of grains	QL	VG	Group visual characteristic, type I
C39	Ear: type of grain	QN	VG	Group visual characteristic, type I
C40	Ear: color of top of grain	PQ	VG	Group visual characteristic, type I
C41	Ear: color of dorsal side of grain	PQ	VG	Group visual characteristic, type I
C42	Kernel shape	PQ	VG	Group visual characteristic, type I
C43	Ear: anthocyanin coloration of glumes of cob	QN	VG	Group visual characteristic, type I
C17	Tassel: length of main axis above lowest lateral branch	QN	MS	Individual measured characteristic
C18	Tassel: length of main axis above highest lateral branch	QN	MS	Individual measured characteristic
C19	Tassel: number of primary lateral branches	QN	MS	Individual measured characteristic
C20	Tassel: length of lateral branch	QN	MS	Individual measured characteristic
C23	Leaf: length of blade	QN	MS	Individual measured characteristic
C24	Leaf: width of blade	QN	MS	Individual measured characteristic
C26.1	Ear height (inbred variety)	QN	MS	Individual measured characteristic
C26.2	Ear height (hybrid variety)	QN	MS	Individual measured characteristic
C27.1	Plant: height (inbred variety)	QN	MS	Individual measured characteristic
C27.2	Plant: height (hybrid variety)	QN	MS	Individual measured characteristic
C28.1	Plant: ratio height of insertion of peduncle of upper ear to plant length (inbred variety)	QN	MS	Individual measured characteristic
C28.2	Plant: ratio height of insertion of peduncle of upper ear to plant length (hybrid variety)	QN	MS	Individual measured characteristic
C30.1	Ear: length (inbred variety)	QN	MS	Individual measured characteristic
C30.2	Ear: length (hybrid variety)	QN	MS	Individual measured characteristic
C31.1	Ear: diameter (inbred variety)	QN	MS	Individual measured characteristic
C31.2	Ear: diameter (hybrid variety)	QN	MS	Individual measured characteristic
C32.1	Ear: number of rows of grain (inbred variety)	QN	MS	Individual measured characteristic
C32.2	Ear: number of rows of grain (hybrid variety)	QN	MS	Individual measured characteristic
C4	Tassel: time of anthesis	QN	MG	Group measured characteristic
C5	Ear: time of silk emergence	QN	MG	Group measured characteristic
C6	Leaf: angle between blade and stem	QN	VG	Group visual characteristic, type II
C7	Leaf: curvature of blade	QN	VG	Group visual characteristic, type II
C11	Tassel: density of spikelets	QN	VG	Group visual characteristic, type II
C15	Tassel: angle between main axis and lateral branches	QN	VG	Group visual characteristic, type II
C16	Tassel: curvature of lateral branches	QN	VG	Group visual characteristic, type II
C21	Stem: degree of zig-zag	QN	VG	Group visual characteristic, type II
C29	Peduncle: length	QN	VG	Group visual characteristic, type II

#### 2.3.2 Characteristic code correction

Based on the expression disparities observed between the two locations, the measured characteristics were categorized into two treatment groups: characteristics exhibiting no significant differences between sites directly adopted a unified standard, whereas those demonstrating systemic differences were calibrated by adjusting the grading code standards. This correction process was performed to guarantee the accuracy of the subsequent statistical analysis. T-test indicated significant systematic phenotypic divergence for several characteristics (e.g., C2 and C25) between the two experimental sites (*P* < 0.05). To harmonize grading benchmarks, phenotypic data from the 2025 Datong trial were normalized to the grading scale of the 2025 Xinzhou trial via linear transformation. Importantly, this scale calibration merely standardizes rating criteria to facilitate inter-site data comparison and does not remove inherent genotype-by-environment (G × E) interactions.

## 3 Experimental protocol

### 3.1 Analysis of variation coefficient differences

Mean values and standard deviations for each characteristic were computed across temporal (inter-annual) and spatial (inter-site) dimensions using SPSS software, and the coefficient of variation was derived via the following formula: CV = (standard deviation/mean) × 100%. By comparing the divergence in CV values across these dimensions, the degree of characteristic sensitivity to environmental fluctuations was systematically evaluated.

### 3.2 Principal component analysis

Before performing PCA, quantitative characteristic data were *Z*-score-standardized, to remove the effects of different units of measurement, using squared Euclidean distance between vectors. Matrix calculations were conducted using SPSS or NTSYSpc software. Components with eigenvalues >1 were retained, and their variance contribution rates were calculated to measure the total variation explained. The concentration of variance was further verified by plotting scree plots to observe the decay trend of eigenvalues. The screening of core characteristics relied primarily on factor loading intensities—specifically the vector length and its orientation in the loading plot—to identify highly representative characteristics along PC1 and PC2 while eliminating redundant indicators with highly aligned loading directions, ultimately constructing a precision maize evaluation system.

### 3.3 Cluster analysis

An initial data matrix was first constructed based on the standardized DUS characteristic observations. Subsequently, similarity distance matrices between varieties were computed using Origin software, utilizing squared Euclidean distance as the metric to quantify phenotypic divergence. Regarding algorithmic selection, hierarchical clustering was performed via the unweighted pair-group method with arithmetic mean (UPGMA), and variety clustering dendrograms were generated. The taxonomic stability of phenotypic characteristic under environmental fluctuations was quantitatively evaluated by systematically comparing the topological consistency of clustering branch structures across different sites and years.

### 3.4 Criteria for distinctness assessment

In accordance with the principles specified in UPOV Technical Guidance Document TGP/9 (UPOV, 2019), after completion of a two-location trial within one growing cycle, comparative phenotypic assessment between the candidate variety and the relevant similar variety shall be performed, and distinctness shall be determined under the “1-year, two-location” testing scheme following the rules below:

- The candidate variety is clearly distinguishable from the similar variety at both trial locations with identical distinguishing characteristic and consistent direction of differences: distinctness is confirmed.
- No clear distinction between the candidate and the similar variety is observed at either location: distinctness is not established.
- Distinguishing characteristic and the direction of characteristic differences are identical across both locations, yet the sufficiency of characteristic divergence cannot be verified using data from a single site for at least one location: distinctness is confirmed.
- Two scenarios trigger follow-up trials: (i) Distinguishing characteristic differ between the two locations or the same characteristic(s) show opposing difference directions, with significant divergence observed at both sites; (ii) For the same characteristic(s), a clear difference is detected at one location, whereas no evident divergence exists at the other location. In such cases, successive-cycle trials shall be conducted at the site where significant characteristic differences were detected in the previous cycle. Distinctness is confirmed if the observed characteristic divergence is reproducible and remains significant across two consecutive cycles at the same location; otherwise, distinctness is denied.

## 4 Results and analysis

### 4.1 Evaluation of spatiotemporal trial site applicability and core characteristic screening in maize DUS testing based on coefficient of variation analysis

The coefficient of variation (CV) serves as a pivotal parameter for evaluating the phenotypic dispersion of fundamental maize DUS characteristic and deciphering the driving mechanisms of environmental response. Based on the experimental configuration, the threshold for characteristic stability was established at 30% (CV < 30%): a characteristic was classified as stable when both CV values in the comparison group remained below 30%, whereas it was deemed unstable if either CV reached or exceeded this limit (Yao et al., 2025). Following this criterion, an in-depth comparative analysis was conducted for “Xinzhou 2025 vs. Datong 2025” (spatial dimension) (**Table 3**) and “Xinzhou 2025 vs. Xinzhou 2024” (temporal dimension) (**Table 4**) to elucidate characteristic expression patterns across heterogeneous environments.

**Table 3:** Phenotypic characteristic description and CV analysis of research materials in spatial experimental sites.

Characteristic number	Characteristic type	Observation method	Characteristic codes, Xinzhou site, 2025				Characteristic codes, Datong site, 2025				
			Max.	Min.	Coverage area	Coefficient of variation	Max.	Min.	Coverage area	Coefficient of variation	
C1	PQ	VG	4	3	3–4	14%	4	3	3–4	14%	Spatial stability
C2	QN	VG	8	1	1–8	29%	8	1	1–8	29%	Spatial stability
C3	QN	VG	3	2	2–3	10%	3	2	2–3	6%	Spatial stability
C4	QL	VG	8	4	4–8	14%	9	4	4–9	17%	Spatial stability
C5	QN	VG	8	4	4–8	14%	9	4	4–9	16%	Spatial stability
C6	QN	VG	8	2	2–8	28%	6	2	2–6	26%	Spatial stability
C7	QN	VG	8	1	1–8	47%	7	1	1–7	49%	Spatial instability
C8	QN	VG	9	1	1–9	134%	9	1	1–9	129%	Spatial instability
C9	QN	VG	6	1	1–6	53%	6	1	1–6	51%	Spatial instability
C10	QN	VG	8	1	1–8	58%	7	1	1–7	54%	Spatial instability
C11	QN	VG	6	3	3–6	15%	6	3	3–6	17%	Spatial stability
C12	QN	VG	8	1	1–8	102%	8	1	1–8	106%	Spatial instability
C13	QL	VG	8	1	1–8	37%	8	1	1–8	40%	Spatial instability
C14	QN	VG	8	1	1–8	67%	7	1	1–7	72%	Spatial instability
C15	PQ	VG	7	1	1–7	30%	8	2	2–8	28%	Spatial instability
C16	PQ	VG	5	1	1–5	40%	5	1	1–5	31%	Spatial instability
C17	PQ	VG	72.85	24.14	24.14–72.85	15%	48.12	22.45	22.45–48.12	13%	Spatial stability
C18	QN	VG	53.51	16.14	16.14–53.51	16%	36.42	16.24	16.24–36.42	14%	Spatial stability
C19	QN	MS	42.6	1.94	1.94–42.6	60%	27.9	1.25	1.25–27.9	65%	Spatial instability
C20	QN	MS	47.40	15.06	15.06–47.40	18%	34.43	13.9	13.9–34.43	18%	Spatial stability
C21	QN	MS	2	1	1–2	13%	8	1	1–2	9%	Spatial stability
C22	QN	MS	9	1	1–9	66%	8	1	1–9	68%	Spatial instability
C23	QN	MS	102.5	14.25	14.25–102.5	10%	115.5	48.82	48.82–115.5	12%	Spatial stability
C24	QN	MS	14.25	7.31	7.31–14.25	11%	14.56	7.24	7.24–14.56	11%	Spatial stability
C25	QN	MS	2	1	1–2	25%	2	1	1–2	22%	Spatial stability
C26.1	QN	MS	111.4	29.3	29.3–111.4	18%	118.5	32	32–118.5	22%	Spatial stability
C26.2	QN	MS	161	68.25	68.25–161	17%	190.6	74.2	74.2–190.6	17%	Spatial stability
C27.1	QN	MS	195.9	144.1	144.1–195.9	14%	289.6	150.8	150.8–289.6	14%	Spatial stability
C27.2	QN	MS	376.2	196.2	196.2–376.2	16%	388.2	206.4	206.4–388.2	13%	Spatial stability
C28.1	QN	MS	0.45	0.21	0.21–0.45	12%	0.48	0.21	0.21–0.84	15%	Spatial stability
C28.2	QN	MS	0.51	0.29	0.29–0.51	11%	0.55	0.30	0.30–0.55	12%	Spatial stability
C29	QN	MS	4	1	1–4	23%	3	2	2–3	20%	Spatial stability
C30.1	QN	MS	18.84	12.64	12.64–18.84	9%	17.7	13.09	13.09–17.7	8%	Spatial stability
C30.2	QN	MS	25.21	16.14	16.14–25.21	8%	26.4	16.8	16.8–26.4	8%	Spatial stability
C31.1	QN	MS	5.43	3.73	3.73–5.43	10%	5.62	3.71	3.71–5.62	11%	Spatial stability
C31.2	QN	MS	6.39	4.27	4.27–6.39	6%	6.39	4.55	4.55–6.39	5%	Spatial stability
C32.1	QN	MG	19.4	12.9	12.9–19.4	9%	19.3	13.1	13.1–19.3	11%	Spatial stability
C32.2	QN	MG	21	13.4	13.4–21	9%	21.74	13.2	13.2–21.74	9%	Spatial stability
C33	QN	VG	3	1	1–3	8%	3	1	1–3	7%	Spatial stability
C34	QN	VG	3	1	1–3	42%	3	1	1–3	42%	Spatial instability
C39	QN	VG	10	1	1–10	46%	10	1	1–10	43%	Spatial instability
C40	QN	VG	8	1	1–8	45%	8	1	1–8	46%	Spatial instability
C41	QN	VG	8	1	1–8	37%	8	1	1–8	37%	Spatial instability
C42	QN	VG	5	3	3–5	13%	5	3	3–5	14%	Spatial stability
C43	QN	VG	8	1	1–8	63%	9	1	1–9	62%	Spatial instability

**Table 4:** Phenotypic characteristic description and CV analysis of research materials in temporal experimental sites.

Character number	Characteristic type	Observation method	Characteristic codes, Xinzhou Site, 2025				Characteristic codes, Xinzhou Site, 2024				
			Max.	Min.	Coverage area	Coefficient of variation	Max.	Min.	Coverage area	Coefficient of variation	
C1	PQ	VG	4	3	3–4	14%	4	2	2–4	15%	Temporal stability
C2	QN	VG	8	1	1–8	29%	8	1	1–8	31%	Temporal instability
C3	QN	VG	3	2	2–3	10%	3	2	2–3	11%	Temporal stability
C4	QL	VG	8	4	4–8	14%	8	3	3–8	16%	Temporal stability
C5	QN	VG	8	4	4–8	14%	8	3	3–8	15%	Temporal stability
C6	QN	VG	8	2	2–8	28%	6	2	2–6	24%	Temporal stability
C7	QN	VG	8	1	1–8	47%	7	1	1–7	40%	Temporal instability
C8	QN	VG	9	1	1–9	134%	9	1	1–9	130%	Temporal instability
C9	QN	VG	6	1	1–6	53%	6	1	1–6	49%	Temporal instability
C10	QN	VG	8	1	1–8	58%	8	1	1–8	54%	Temporal instability
C11	QN	VG	6	3	3–6	15%	7	3	3–7	17%	Temporal stability
C12	QN	VG	8	1	1–8	102%	9	1	1–9	108%	Temporal instability
C13	QL	VG	8	1	1–8	37%	8	1	1–8	39%	Temporal instability
C14	QN	VG	8	1	1–8	67%	8	1	1–8	76%	Temporal instability
C15	PQ	VG	7	1	1–7	30%	9	1	1–9	35%	Temporal instability
C16	PQ	VG	5	1	1–5	40%	5	1	1–5	38%	Temporal instability
C17	PQ	VG	72.85	24.14	24.14–72.85	15%	48.12	22.25	22.25–48.12	12%	Temporal stability
C18	QN	VG	53.51	16.14	16.14–53.51	16%	41.37	15.77	15.77–41.37	15%	Temporal stability
C19	QN	MS	42.6	1.94	1.94–42.6	60%	31.05	1.35	1.35–31.05	73%	Temporal instability
C20	QN	MS	47.40	15.06	15.06–47.40	18%	41.14	10.82	10.82–41.14	20%	Temporal stability
C21	QN	MS	2	1	1–2	13%	2	1	1–2	13%	Temporal stability
C22	QN	MS	9	1	1–9	66%	9	1	1–9	57%	Temporal instability
C23	QN	MS	102.5	14.25	14.25–102.5	10%	102.10	60.17	60.17–102.1	10%	Temporal stability
C24	QN	MS	14.25	7.31	7.31–14.25	11%	13.79	6.94	6.94–13.79	12%	Temporal stability
C25	QN	MS	2	1	1–2	25%	3	1	1–3	46%	Temporal instability
C26.1	QN	MS	111.4	29.3	29.3–111.4	18%	126.3	41.2	41.2–126.3	22%	Temporal stability
C26.2	QN	MS	161	68.25	68.25–161	17%	149.5	31.95	31.95–149.5	18%	Temporal stability
C27.1	QN	MS	195.9	144.1	144.1–195.9	14%	293.2	158.7	158.7–293.2	13%	Temporal stability
C27.2	QN	MS	376.2	196.2	196.2–376.2	16%	359.7	146.7	146.7–359.7	14%	Temporal stability
C28.1	QN	MS	0.45	0.21	0.21–0.45	12%	0.45	0.19	0.19–0.45	15%	Temporal stability
C28.2	QN	MS	0.51	0.29	0.29–0.51	11%	0.48	0.22	0.22–0.48	11%	Temporal stability
C29	QN	MS	4	1	1–4	23%	4	2	2–4	21%	Temporal stability
C30.1	QN	MS	18.84	12.64	12.64–18.84	9%	20	2.42	2.42–20	15%	Temporal stability
C30.2	QN	MS	25.21	16.14	16.14–25.21	8%	25.96	2.25	2.25–25.96	38%	Temporal instability
C31.1	QN	MS	5.43	3.73	3.73–5.43	10%	5.77	4	4–5.77	8%	Temporal stability
C31.2	QN	MS	6.39	4.27	4.27–6.39	6%	6.25	4.50	4.5–6.25	6%	Temporal stability
C32.1	QN	MG	19.4	12.9	12.9–19.4	9%	18.9	2.9	2.9–18.9	14%	Temporal stability
C32.2	QN	MG	21	13.4	13.4–21	9%	21.1	2.1	2.1–21.1	1%7	Temporal stability
C33	QN	VG	3	1	1–3	8%	3	1	1–3	9%	Temporal stability
C34	QN	VG	3	1	1–3	42%	3	1	1–3	44%	Temporal instability
C39	QN	VG	10	1	1–10	46%	10	1	1–10	46%	Temporal instability
C40	QN	VG	8	1	1–8	45%	8	1	1–8	43%	Temporal instability
C41	QN	VG	8	1	1–8	37%	9	1	1–9	38%	Temporal instability
C42	QN	VG	5	3	3–5	13%	5	3	3–5	14%	Temporal stability
C43	QN	VG	8	1	1–8	63%	9	1	1–8	63%	Temporal instability

Visual-group characteristics (VG), which primarily encompass the morphological features and qualitative descriptions of maize, provide a fundamental basis for the intuitive determination of variety distinctness in DUS testing. Characteristics 1, 3, 6, 11, 21, 29, 33, and 42 exhibited remarkable genetic robustness across both spatial and temporal points. Taking C1 as an example, its coefficient of variation at temporal and spatial points ranged from 14% to 15%, indicating an extremely low state of variation convergence. This reveals that such characteristic exhibit highly consistent phenotypic expression across different geographical regions. In contrast, characteristics 7, 8, 9, 10, 12, 13, 14, 15, 16, 22, 34, 39, 40, 41, and 43 were unstable at both temporal and spatial points. Notably, characteristics 8 and 12 showed coefficients of variation exceeding 100% at both temporal points. Such an extremely high degree of dispersion reflects substantial phenotypic differences within the population for these characteristic, as well as their high susceptibility to induced fluctuations by microenvironmental factors. Temporally, the stability of VG characteristic faces severe challenges from stochastic annual climatic noise. While C1 and C3–C6 maintained temporal stability, C2 exhibited significant temporal sensitivity; its CV value at spatial points was uniformly 29%, while the CV value at temporal points ranged from 29% to 31%—thus, falling into the temporally unstable category according to our decision rules. This year-to-year variation suggests that while the expression of C2 is spatially uniform, it is sensitive to annual weather factors such as precipitation or accumulated temperature. Overall, only approximately 30% of VG characteristic achieved stability across both spatial and temporal dimensions, necessitating the calibration of inter-annual discrepancies during DUS examinations for these indicators.

For measured group (MG) and measured individual (MS) characteristic, this study compared the phenotypic performance of inbred lines (CS) and hybrids (CH) to reveal differences in environmental response under various genetic backgrounds. Within the inbred line (CS) group, measured characteristic demonstrated exceptional phenotypic expressivity and statistical robustness. Characteristics 4, 5, 17, 18, 20, 23, 24, 26.1, 27.1, 28.1, 30.1, 31.1, and 32.1 constituted a core stability cluster, with CV values across both dimensions significantly below 30%—for instance, the spatial CVs for C31.1 were 10% and 11%, further converging to 8% temporally, suggesting that these quantitative indicators in inbred lines are tightly regulated by potent genes and possess formidable resistance to environmental perturbation. As an MG characteristic, the spatiotemporal CV of C32.1 fluctuated only between 9% and 14%, effectively harnessing the smoothing effect of population averages against stochastic noise. Conversely, in maize hybrids (CH), characteristic C4 through C32.2 exhibited stability across all dimensions, with CV values generally remaining below 20%. Nevertheless, C30.2 displayed extreme inter-annual volatility: while spatially ultra-stable in 2025 (CV = 8%), its temporal CV surged to 38% in 2024, manifesting a pronounced pattern of temporal instability. Furthermore, characteristic C19 (spatial CV up to 65%) and C22 (spatial CV up to 68%) exhibited severe fluctuations across all dimensions, underscoring the extreme sensitivity of these MS indicators to environmental conditions and rendering them unsuitable as core references for uniformity assessment. Simultaneously, characteristic C25 characterized by “spatial stability (25%/22%) vs. temporal instability (46%)” further validates that temporal pressure constitutes a central challenge to the precision of individual measurements.

In summary, through a quantitative analysis of 39 characteristics, this study further deepens the understanding of the expression patterns of maize DUS characteristic across both temporal and spatial dimensions. Given that most core measured indicators exhibit robust spatiotemporal stability, this research suggests that integrating a multi-site spatial repetition model as a powerful supplement to traditional temporal testing holds substantial scientific value. This synergistic spatiotemporal observation mode achieves broader coverage of environmental variation via increased spatial data points, effectively mitigating random weather interference for characteristics like C30.2 or C25 during particular years. By effectively supplementing the temporal dimension with spatial sites, we can provide a richer decision-making basis for optimizing DUS testing cycles and enhancing substantive examination efficiency while ensuring the accuracy of variety determination.

### 4.2 Construction of maize phenotypic observation scheme based on spatiotemporal stability of characteristic and evaluation of its application value

In the scientific practice of maize phenotypic identification and variety evaluation, an in-depth dissection of characteristic stability across diverse spatiotemporal dimensions forms the core foundation for constructing efficient and precise observation systems. By analyzing differences across characteristics in maize inbred lines and hybrids, the regulatory logic of genotype-by-environment (G × E) interactions on phenotypic expression can be clearly revealed (Kusmec et al., 2017; Silveira et al., 2024; Tibbs-Cortes et al., 2024).

Among visual group (VG) characteristics (**Figures 1A, B**), C1 through C43 (including C2, C6, C7, etc.) showed no significant differences across spatiotemporal points, indicating minimal variation for these characteristics. Notably, C3 and C6 exhibited no significant spatial differences, while C42 remained stable temporally, whereas C15 and C29 demonstrated significant differences across all spatiotemporal points. Analysis of measured group (MG) and measured individual (MS) characteristics (**Figure 1C**) revealed that within common maize inbred lines (CS), C5, C18, C23, C27.1, and C31.1 remained consistent across all spatiotemporal points, while C20 showed significant spatial variation, C19 through C32.1 exhibited significant temporal shifts, and C4 and C17 displayed significant differences in both dimensions. In common maize hybrids (CH), C5, C18, and C23 maintained stability across spatiotemporal points, with C20, C28.2, and C30.2 showing no significant spatial variation and C19 and C24 remaining temporally stable. Conversely, C4, C17, C26.2, C27.2, C31.2, and C32.2 all exhibited significant spatiotemporal differences. In summary, the findings indicate that spatiotemporal difference analysis based on significance testing effectively distinguishes characteristic sensitivity, providing a vital statistical framework for stability evaluation, core characteristic selection, and test site optimization in maize DUS testing.

**Figure 1:**
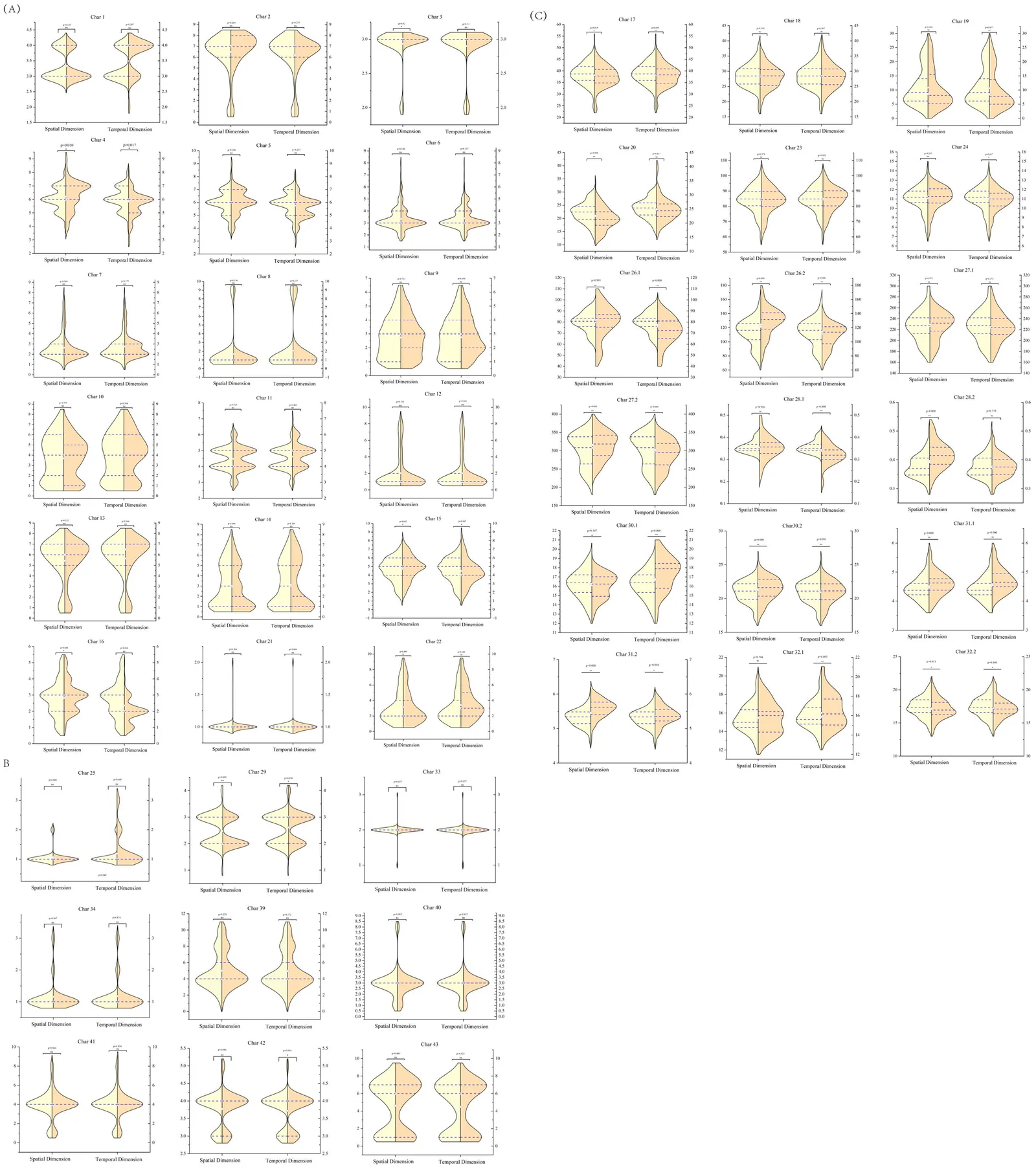
**(A, B)** Comparison of phenotypic data distribution of visual characteristic at spatial and temporal sites. **(C)** Comparison of phenotypic data distribution of measured characteristic at spatial and temporal sites. Note: * P < 0.05, ** P < 0.01; ns, not significant.

### 4.3 Analysis of spatiotemporal stability of maize DUS characteristic based on PCA and construction of core evaluation system

Building upon the CV-based quantitative analysis and qualitative classification of spatiotemporal stability, we further employed principal component analysis (PCA) to perform dimensionality reduction on the tested characteristic, revealing the intrinsic structural characteristics of maize phenotypic variation within a multidimensional space. The PCA results not only validated the prior conclusions regarding characteristic environmental sensitivity but also leveraged the distinct characteristics of eigenvalues and variance contribution rates to provide a multivariate scientific basis for screening core maize DUS characteristic (Palaniyappan et al., 2023; Uddin et al., 2023; Shen et al., 2025).

First, the variation-resolving power of PCA corroborated the dominant regulatory role of the spatial environment on the maize phenotypic structure. Research indicated that the cumulative variance contribution of the first 12 principal components in the spatial dimension reached 74.92%, which was significantly higher than the 67.50% achieved by the first 11 principal components in the temporal dimension (**Tables 5**, **6**). Second, the PCA score plots illustrated that the 95% confidence ellipses for different test sites and years exhibited high levels of overlap. This distribution pattern indicates that despite the presence of sensitive indicators like characteristic 2 and 25, the overall phenotypic genetic expression structure of the varieties underwent no fundamental shift, which logically complements the large group of stable characteristic identified (e.g., characteristics 1, 3, 4, 5, 6, 11, 17, 18, 20, and 21, all satisfying CV <30%). Third, the selection of core characteristic based on principal component loading intensity enabled the streamlining of stable characteristic groups and the assignment of appropriate weights. The PCA results demonstrated that C27 (plant height) exhibited the maximum loading value in the direction of the first principal component (PC1) across both dimensions, identifying it as a core indicator for differentiating variety phenotypic variation. Simultaneously, C27, C41, C39, C9, C23, C18, and C13 were screened as core characteristic based on their loading intensities (**Figures 2A–F**).

**Table 5:** PCA of DUS characteristics across spatial points.

Principal component number	Eigenvalue	Percentage of variance (%)	Cumulative (%)
1	6.38869	16.38126	16.38126
2	5.14201	13.18465	29.56591
3	3.44374	8.83011	38.39602
4	2.71405	6.95911	45.35513
5	2.1257	5.45051	50.80564
6	1.88496	4.83323	55.63888
7	1.77237	4.54454	60.18342
8	1.28616	3.29784	63.48126
9	1.22918	3.15174	66.633
10	1.16815	2.99526	69.62826
11	1.0352	2.65435	72.28261
12	1.02675	2.63268	74.91529
13	0.94407	2.42069	77.33598
14	0.79319	2.03382	79.3698
15	0.70341	1.80363	81.17343
16	0.61403	1.57444	82.74787
17	0.60317	1.54659	84.29446
18	0.57751	1.4808	85.77526
19	0.52013	1.33367	87.10893
20	0.50087	1.28428	88.39321
21	0.484	1.24103	89.63424
22	0.43485	1.11499	90.74923
23	0.41343	1.06008	91.80932
24	0.39168	1.0043	92.81362
25	0.33621	0.86208	93.6757
26	0.32297	0.82813	94.50383
27	0.30234	0.77524	95.27907
28	0.26846	0.68837	95.96744
29	0.26013	0.667	96.63443
30	0.24915	0.63885	97.27328
31	0.21292	0.54595	97.81924
32	0.19818	0.50815	98.32738
33	0.19241	0.49336	98.82074
34	0.13994	0.35881	99.17955
35	0.12619	0.32357	99.50312
36	0.08348	0.21406	99.71717
37	0.05242	0.13441	99.85158
38	0.04382	0.11235	99.96394
39	0.01406	0.03606	100

**Table 6:** PCA of DUS characteristic across time points.

Principal component number	Eigenvalue	Percentage of variance (%)	Cumulative (%)
1	5.86991	15.05104	15.05104
2	4.7059	12.06642	27.11747
3	3.05546	7.83451	34.95197
4	2.41645	6.19601	41.14799
5	2.18247	5.59607	46.74406
6	1.7191	4.40794	51.152
7	1.60001	4.10259	55.25459
8	1.32663	3.40163	58.65621
9	1.20974	3.1019	61.75812
10	1.17794	3.02035	64.77847
11	1.06247	2.72428	67.50276
12	0.9777	2.50692	70.00967
13	0.97015	2.48757	72.49724
14	0.92495	2.37167	74.86891
15	0.87203	2.23598	77.10489
16	0.77842	1.99596	79.10085
17	0.74222	1.90313	81.00398
18	0.72486	1.85863	82.86261
19	0.6657	1.70692	84.56953
20	0.64323	1.64931	86.21884
21	0.55548	1.4243	87.64314
22	0.53414	1.36959	89.01274
23	0.47382	1.21493	90.22767
24	0.4404	1.12924	91.35691
25	0.38558	0.98865	92.34556
26	0.36958	0.94764	93.29321
27	0.36614	0.93882	94.23202
28	0.35891	0.92028	95.15231
29	0.3351	0.85924	96.01154
30	0.28846	0.73964	96.75119
31	0.26904	0.68984	97.44103
32	0.24322	0.62363	98.06466
33	0.23452	0.60134	98.666
34	0.17015	0.43627	99.10227
35	0.14761	0.37848	99.48075
36	0.09659	0.24766	99.72841
37	0.05808	0.14893	99.87733
38	0.04071	0.10437	99.98171
39	0.00713	0.01829	100

**Figure 2:**
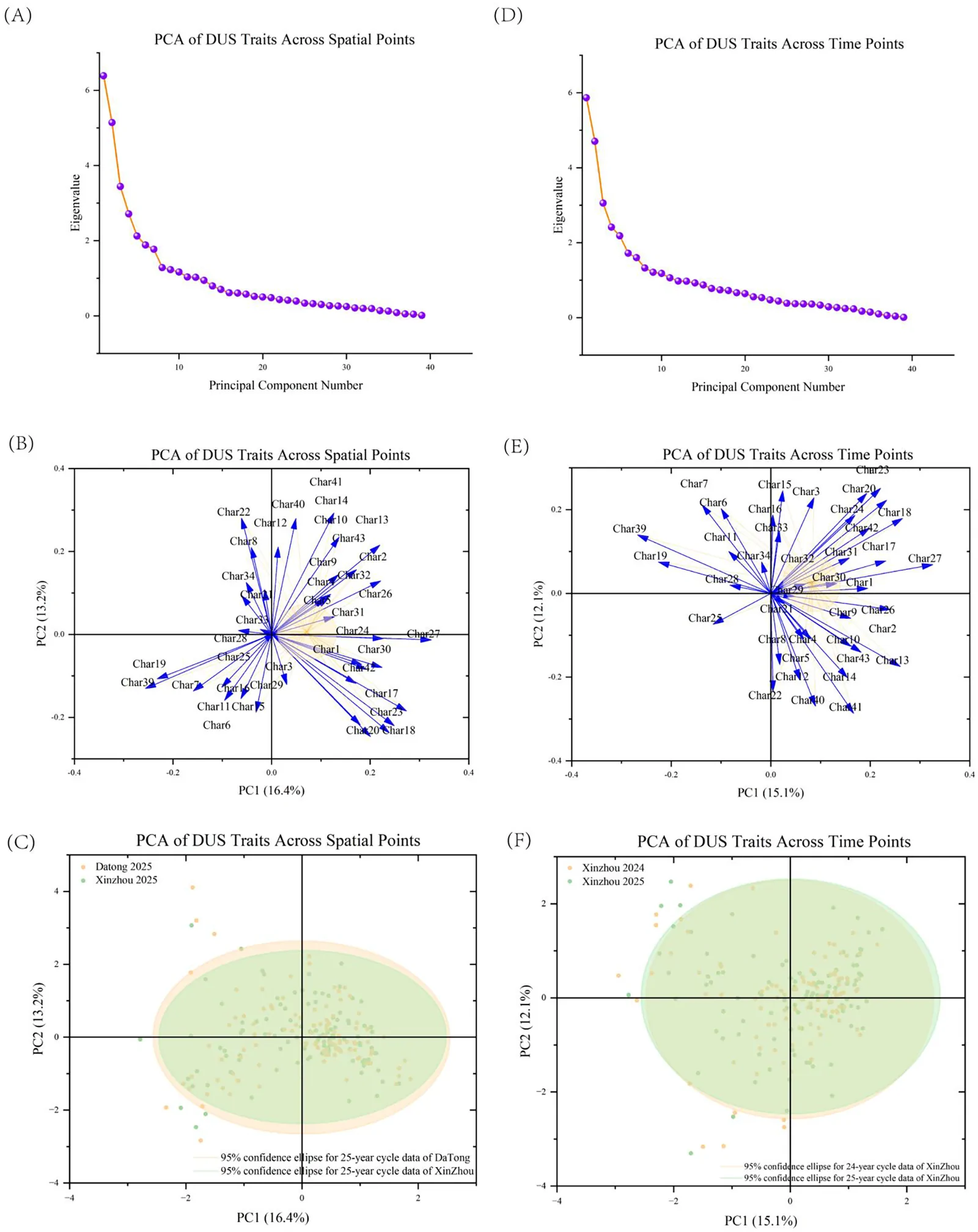
**(A–C)** PCA of DUS characteristic across spatial points. **(D–F)** PCA of DUS characteristic across time points.

### 4.4 Comprehensive evaluation of spatiotemporal stability of maize DUS characteristic based on cluster membership consistency rate

By comparing the variety clustering results between two spatial sites (Xinzhou and Datong in 2025) and two temporal points (Xinzhou in 2024 and 2025), it was observed that the clustering retention (CR) rate reached 92.31% in the spatial dimension (with nine varieties, including 2021101338B and others, failing to cluster together) (**Figures 3A, C**), while the temporal CR rate was even higher at 94.87% (with six varieties, such as 2021101338B and others, ungrouped) (**Figures 3B, D**). This robust consistency exceeding 92% across diverse spatiotemporal conditions validates the scientific feasibility and high stability of utilizing spatial sites for variety clustering. Further analysis indicated that the rare instances of clustering inconsistency primarily stem from environmentally induced differences in phenotypic plasticity. These varieties frequently harbor spatiotemporally sensitive characteristic, whose expression is finely modulated by environmental factors such as geographic location, soil fertility, and annual climatic fluctuations, leading to shifts at the peripheral boundaries of dendrogram clusters. This result is highly consistent with the earlier selection of sensitive characteristic based on CV, confirming that environmental factors only slightly adjust the clustering pattern rather than dominating it.

**Figure 3:**
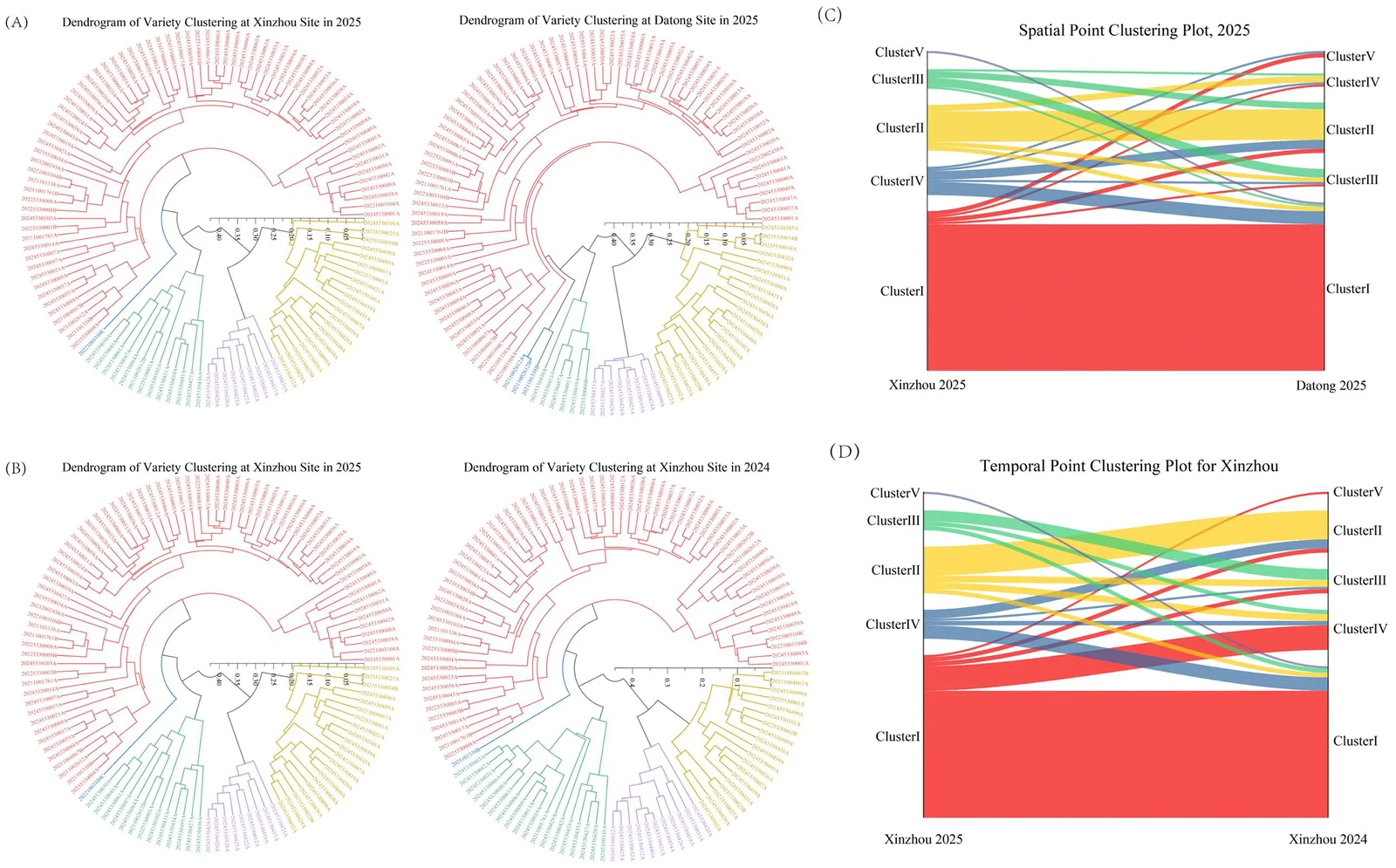
**(A)** Variety clustering results at spatial sites. **(B)** Variety clustering results at time sites. **(C)** Spatial point clustering plot, 2025. **(D)** Temporal point clustering plot for Xinzhou.

### 4.5 Verification of core effectiveness of spatial replication patterns based on spatiotemporal comparison in the determination of distinctness and uniformity of maize varieties

Based on the PCA and CR results, 106 maize accessions were evaluated to verify the effectiveness of the spatial replication mode for DUS distinctness testing. The efficacy analysis result revealed (**Table 7**) that the distinctness consistency rate between spatial sites reached 94.36%, significantly outperforming the 86.79% observed in cross-year temporal trials. The detailed analysis showed that some hybrids were sensitive to annual weather variation (e.g., rainfall and temperature), leading to inconsistent distinctness results across years, while the results remained stable across locations in the same year (**Figures 4A, B**). This confirms that the spatial replication mode reduces the impact of G × E interactions better than the temporal mode, improving the reliability of Distinctness testing and supporting the earlier conclusion that spatial trials provide better variation analysis.

**Table 7:** Comparison of specificity test results between spatial and temporal maize experiments.

Test code	Time points		Spatial points	
	2025, Xinzhou	2024, Xinzhou	2025, Xinzhou	2025, Datong
20245330001A	○	√	○	○
20245330002A	√	√	√	√
20245330003A	√	√	√	√
20245330004A	√	√	√	√
20245330005A	√	√	√	√
20245330006A	√	√	√	√
20245330007A	√	√	√	√
20245330009A	○	√	○	○
20245330010A	√	√	√	√
20245330011A	√	√	√	√
20245330012A	√	√	√	√
20245330013A	√	√	√	√
20245330014A	√	√	√	√
20245330015A	√	√	√	√
20245330016A	○	√	○	○
20245330017A	√	√	√	√
20245330018A	√	√	√	√
20245330019A	○	√	○	○
20245330020A	√	√	√	√
20245330021A	√	√	√	√
20245330023A	√	√	√	√
20245330024A	√	√	√	√
20245330026A	√	√	√	√
20245330028A	√	√	√	√
20245330030A	√	√	√	√
20245330031A	√	√	√	√
20245330034A	√	√	√	√
20245330036A	√	√	√	√
20245330037A	√	√	√	√
20245330038A	√	√	√	√
20245330039A	√	√	√	√
20245330040A	√	√	√	√
20245330041A	√	√	√	√
20245330042A	√	√	√	√
20245330043A	√	√	√	√
20245330044A	√	√	√	√
20245330045A	√	√	√	√
20245330046A	√	√	√	√
20245330047A	√	√	√	√
20245330048A	√	√	√	√
20245330049A	√	√	√	√
20245330050A	√	√	√	√
20245330051A	√	√	√	√
20245330053A	√	√	√	√
20245330054A	√	√	√	√
20245330055A	√	√	√	√
20245330056A	○	√	○	○
20245330057A	√	√	√	√
20245330058A	√	√	√	√
20245330059A	√	√	√	√
20245330061A	√	√	√	√
20245330062A	√	√	√	√
20245330063A	√	√	√	√
20245330064A	√	√	√	√
20245330065A	√	√	√	√
20245330066A	√	√	√	√
20245330067A	√	√	√	√
20245330423A	√	√	√	√
20245330424A	√	√	√	√
20245330422A	√	√	√	√
20245330432A	√	√	√	√
20245330102A	√	√	√	√
20245330429A	√	√	√	√
20245330430A	√	√	√	√
20245330431A	√	√	√	√
20245330433A	√	√	√	√
20245330434A	√	√	√	√
20245330435A	√	√	√	√
20245330438A	√	√	√	√
20245330439A	√	√	√	√
20245330489A	√	√	√	√
20245330490A	√	√	√	√
20245330099A	○	√	○	○
20245330426A	√	√	√	√
20245330425A	√	√	√	√
20245330418A	√	√	√	√
20245330440A	√	√	√	√
20245330419A	○	○	○	○
20245330436A	√	√	√	√
20245330345A	√	√	√	√
20245330101A	√	√	√	√
20245330420A	√	√	√	○
20245330487A	√	√	√	√
20245330491A	√	√	√	√
20245330427A	√	√	√	√
20245330428A	√	√	√	√
20245330032A	√	√	√	√
20245330033A	×	√	×	○
20245330035A	○	√	○	√
20245330052A	√	√	√	√
20245330060A	√	√	√	√
20245330103A	√	√	√	√
20245330437A	√	√	√	√
20245330421A	√	√	√	√
20225330081A	×	×	×	×
20235330034A	√	×	√	√
20235330025A	×	×	×	×
20211004867A	×	×	×	×
20211002612A	√	×	√	√
20232002438A	×	×	×	○
20221003104A	○	×	○	√
2021101338A	×	×	×	○
20235330004A	√	×	√	√
20225330003A	√	×	√	√
20211001761A	×	○	×	×
20225330008A	×	×	×	×

**Figure 4:**
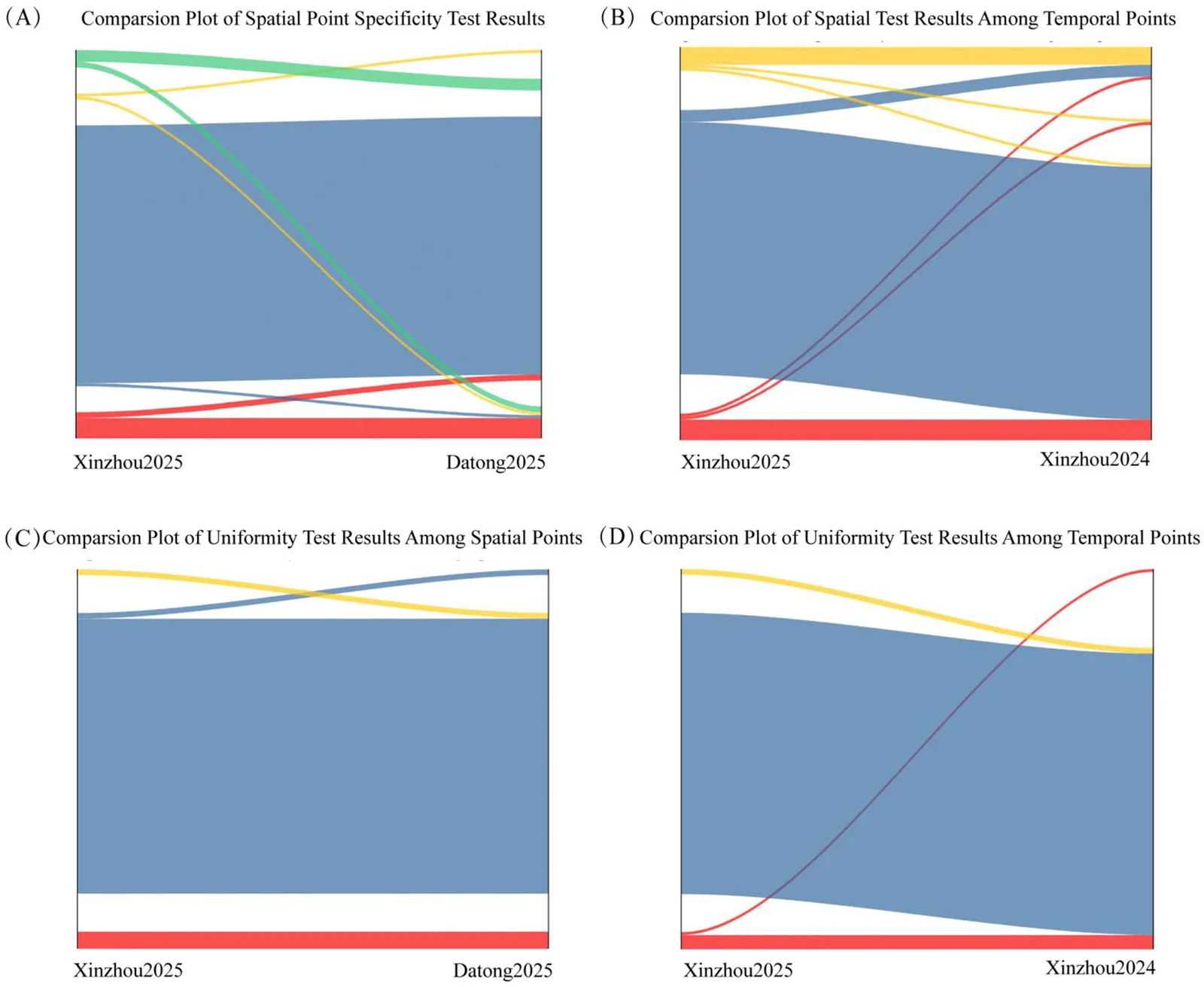
**(A)** Comparison plot of spatial point specificity test results. **(B)** Comparison plot of specificity test results among temporal points. **(C)** Comparison plot of uniformity test results among spatial points. **(D)** Comparison plot of uniformity test results among temporal points.

The analysis of uniformity determination further corroborated the superiority of the spatial replication model (**Table 8**). The results showed that the consistency for uniformity was 96.23% between locations, better than 93.4% across years. The inconsistency found in multi-year trials for certain varieties was effectively corrected in spatial trials (**Figures 4C, D**). The PCA results offered technical support for these findings: the first 12 components in the spatial dimension explained 74.92% of the variance, and samples from different locations overlapped significantly in the PCA plots. This distribution pattern validates that the holistic phenotypic framework of maize varieties maintains exceptional robustness during spatial shifts and remains free from fundamental interference by environmental heterogeneity, which is the intrinsic reason why the spatial replication model ensures diagnostic accuracy. In summary, the promotion of the “1-year, multi-location” spatial replication model, grounded in characteristic stability, successfully achieves a rational reduction in testing cycles while ensuring scientific rigor, providing a formidable empirical basis for enhancing the efficiency of substantive examinations and supporting the revitalization of the seed industry in China.

**Table 8:** Comparison of uniformity test results between spatial and temporal maize experiments.

Test code	Time points		Spatial points	
	2025, Xinzhou	2024, Xinzhou	2025, Xinzhou	2025, Datong
20245330001A	×	√	×	×
20245330002A	√	√	√	√
20245330003A	√	√	√	√
20245330004A	√	√	√	√
20245330005A	√	√	√	√
20245330006A	√	√	√	√
20245330007A	√	√	√	√
20245330009A	×	√	×	×
20245330010A	√	√	√	√
20245330011A	√	√	√	√
20245330012A	√	√	√	√
20245330013A	√	√	√	√
20245330014A	√	√	√	√
20245330015A	√	√	√	√
20245330016A	×	√	×	×
20245330017A	√	√	√	√
20245330018A	√	√	√	√
20245330019A	×	√	×	×
20245330020A	√	√	√	√
20245330021A	√	√	√	√
20245330023A	√	√	√	√
20245330024A	√	√	√	√
20245330026A	√	√	√	√
20245330028A	√	√	√	√
20245330030A	√	√	√	√
20245330031A	√	√	√	√
20245330034A	√	√	√	√
20245330036A	√	√	√	√
20245330037A	√	√	√	√
20245330038A	√	√	√	√
20245330039A	√	√	√	√
20245330040A	√	√	√	√
20245330041A	√	√	√	√
20245330042A	√	√	√	√
20245330043A	√	√	√	√
20245330044A	√	√	√	√
20245330045A	√	√	√	√
20245330046A	√	√	√	√
20245330047A	√	√	√	√
20245330048A	√	√	√	√
20245330049A	√	√	√	○
20245330050A	√	√	√	√
20245330051A	√	√	√	√
20245330053A	√	√	√	√
20245330054A	√	√	√	√
20245330055A	√	√	√	√
20245330056A	×	√	×	×
20245330057A	√	√	√	√
20245330058A	√	√	√	√
20245330059A	√	√	√	√
20245330061A	√	√	√	√
20245330062A	√	√	√	√
20245330063A	√	√	√	√
20245330064A	√	√	√	√
20245330065A	√	√	√	√
20245330066A	√	√	√	√
20245330067A	√	√	√	√
20245330423A	√	√	√	√
20245330424A	√	√	√	√
20245330422A	√	√	√	√
20245330432A	√	√	√	√
20245330102A	√	√	√	√
20245330429A	√	√	√	√
20245330430A	√	√	√	√
20245330431A	√	√	√	√
20245330433A	√	√	√	√
20245330434A	√	√	√	√
20245330435A	√	√	√	√
20245330438A	√	√	√	√
20245330439A	√	√	√	√
20245330489A	√	√	√	√
20245330490A	√	√	√	√
20245330099A	√	√	√	√
20245330426A	√	√	√	√
20245330425A	√	√	√	√
20245330418A	√	√	√	√
20245330440A	√	√	√	√
20245330419A	×	×	×	×
20245330436A	√	√	√	√
20245330345A	○	√	○	√
20245330101A	√	√	√	√
20245330420A	√	√	√	√
20245330487A	√	√	√	√
20245330491A	√	√	√	√
20245330427A	√	√	√	√
20245330428A	√	√	√	√
20245330032A	√	√	√	√
20245330033A	√	√	√	√
20245330035A	√	√	√	√
20245330052A	√	√	√	√
20245330060A	√	√	√	√
20245330103A	√	√	√	√
20245330437A	○	√	○	√
20245330421A	√	√	√	√
20225330081A	√	√	√	√
20235330034A	√	√	√	√
20235330025A	√	√	√	○
20211004867A	√	√	√	√
20211002612A	√	√	√	√
20232002438A	√	√	√	√
20221003104A	√	√	√	√
2021101338A	√	√	√	√
20235330004A	√	√	√	√
20225330003A	√	√	√	√
20211001761A	√	√	√	√
20225330008A	√	√	√	√

## 5 Conclusion

By deciphering the patterns of maize phenotypic variation under heterogeneous spatiotemporal environments, this study constructed a complete logical closed-loop—extending from “characteristic response characteristics” to “variety structural robustness” and ultimately to “testing model efficacy enhancement”.

Firstly, the research revealed that fundamental maize DUS characteristic exhibits significant expression differentiation patterns driven by the environment. Through CV-based quantitative screening, a spatiotemporally stable cluster (including C1, C3, C11, etc.) was identified as the core benchmark for variety identification and fingerprinting. Simultaneously, C2, C25, and C30.2 were identified as possessing significant spatial sensitivity, while others such as C7–C10 were subject to dual spatiotemporal regulation by annual meteorological fluctuations, with CV values exceeding 30% in both dimensions.

Secondly, principal component analysis (PCA) deepened the previous single-characteristic findings from a multivariate structural perspective, confirming that while the spatial environment dominates phenotypic variation (74.92% cumulative variance vs. 67.50% temporally), the overall genetic expression framework of the varieties remains robust. Even when a specific sensitive characteristic exhibited phenotypic plasticity, the 95% confidence ellipses for samples across different spatiotemporal sites in the PCA score plots displayed high levels of overlap. This discovery provided a mathematical rationale for optimizing the observation system, enabling the precise selection of an efficient evaluation framework centered on seven core characteristics, such as plant: height (C27), ear: color of dorsal side of grain (C41), and ear: type of grain (C39).

Finally, these theoretical patterns were empirically validated within the spatial repetition testing model: the genetic clustering topology based on characteristic stability maintained a high consistency rate (CR) of 92.31% across different sites. Quantitative efficacy validation demonstrated that the “multi-site in one year” spatial repetition model achieved significantly higher accuracy in both distinctness (94.36%) and uniformity (96.23%) assessments compared to traditional multi-year temporal models. This provides compelling evidence that spatial repetition trials can effectively bypass stochastic noise triggered by annual climatic fluctuations, facilitating a rational shortening of the testing cycle while maintaining the rigor of DUS determinations. In conclusion, this study validates the scientific feasibility of substituting temporal testing with spatial trials based on “environment-insensitive stable characteristic” and “variety structural robustness,” offering a core technical pathway to enhance substantive examination efficiency.

Although this study has elucidated the spatiotemporal stability characteristics and optimization pathways of maize DUS testing characteristic, several avenues remain for deeper exploration in alignment with the evolving demands of the maize seed industry and the frontiers of DUS technology. First, the trial scope and material diversity should be expanded to enhance the universal applicability of the research findings. This study was conducted at two locations in Northern China. However, future trials should include other regions like the Huang-Huai-Hai and Southwest maize zones, alongside more diverse materials (inbreds, hybrids, and local varieties) to ensure that core characteristic stability meets the requirements of national DUS testing standards (Yue et al., 2022; Balconi et al., 2024; Song et al., 2024). Second, the integration of molecular marker technologies is essential to construct a synergistic DUS testing framework merging phenotypic and genotypic data. Since phenotypic characteristics are affected by the environment, combining core characteristic with SSR and SNP markers will help identify stable loci and create models that integrate field observation with molecular identification to improve testing accuracy and efficiency (UPOV, 2002; Gunjaca et al., 2008; Osuman et al., 2022; Akbari et al., 2025). Third, the study of how characteristics respond to the environment should be expanded to improve observation protocols. Although this study provided a preliminary understanding of spatiotemporal regulation, the quantitative associations between specific factors—such as soil fertility, heat sum, and precipitation—and characteristic expression remain relatively underexplored. Future experiments using different environmental gradients can identify thresholds for sensitive characteristic, helping to optimize the timing and location of observations to create a more efficient system that reduces environmental noise (Yang et al., 2024; Bao et al., 2025; He et al., 2025).

## Data Availability

The original contributions presented in the study are included in the article/**Supplementary Material**. Further inquiries can be directed to the corresponding authors.

## References

[B1] Akbari A. Bahmani K. Kazan M. Bilgin Ö.F. Rahimi J. Izadi Darbandi A. (2025). Analysis of fennel breeding populations based on distinctness, uniformity, and stability (DUS) testing via morphological descriptors and DNA molecular markers. Genet. Resour. Crop Evol. 72, 32–342. doi: 10.1007/s10722-024-01934-330311153

[B2] Balconi C. Galaretto A. Malvar R. A. Nicolas S. D. Redaelli R. Andjelkovic V. . (2024). Genetic and phenotypic evaluation of European maize landraces as a tool for conservation and valorization of agrobiodiversity. Biology 13, 454. doi: 10.3390/biology13060454 38927334 PMC11201045

[B3] Bao S. Wang Y. Ma S. Liu H. Xue X. Ma Y. . (2025). Field-scale maize yield estimation using remote sensing with the integration of agronomic characteristic. Agriculture 15, 1834. doi: 10.3390/agriculture1517183430654563

[B4] Chen C. J. Miao Y. H. Wan X. F. Guo L. P. Liu D. H. Huang L. Q. . (2024). Development of DUS testing guidelines of Artemisia argyi. Zhongguo Zhong Yao Za Zhi= Zhongguo Zhongyao Zazhi= China J. Chin. Materia Med. 49, 5835–42. doi: 10.19540/j.cnki.cjcmm.20240718.101 39701793

[B5] Fu R. Wang X. (2023). Modeling the influence of phenotypic plasticity on maize hybrid performance. Plant Commun. 4, 100548. doi: 10.1016/j.xplc.2023.100548 36635964 PMC10203382

[B6] Gunjaca J. Buhinicek I. Jukic M. Sarcevic H. Vragolovic A. Kozic Z. . (2008). Discriminating maize inbred lines using molecular and DUS data. Euphytica 161, 165–172. doi: 10.1007/s10681-007-9518-z30311153

[B7] He K. Yu T. Gao S. Chen S. Li L. Zhang X. . (2025). Leveraging automated machine learning for environmental data‐driven genetic analysis and genomic prediction in maize hybrids. Adv. Sci. 12, 2412423. doi: 10.1002/advs.202412423 40047344 PMC12061318

[B8] Jamnadass R. Mumm R. H. Hale I. Hendre P. Muchugi A. Dawson I. K. . (2020). Enhancing African orphan crops with genomics. Nat. Genet. 52, 356–360. doi: 10.1038/s41588-020-0601-x 32203464

[B9] Jin M. Liu H. Liu X. Guo T. Guo J. Yin Y. . (2023). Complex genetic architecture underlying the plasticity of maize agronomic characteristic. Plant Commun. 4, 100473. doi: 10.1016/j.xplc.2022.100473 36642074 PMC10203269

[B10] Kassem M. A. Knizia D. Meksem K. (2024). A summary of two decades of QTL and candidate genes that control seed tocopherol contents in maize (Zea mays L.). Genes 15, 472. doi: 10.3390/genes15040472 38674406 PMC11049817

[B11] Kusmec A. Srinivasan S. Nettleton D. Schnable P. S. (2017). Distinct genetic architectures for phenotype means and plasticities in Zea mays. Nat. Plants 3, 715–23. doi: 10.1038/s41477-017-0007-7 29150689 PMC6209453

[B12] Li H. Zhang P. Zhang L. Yang J. An X. (2024). Research on the evolutionary characteristics and mechanism of production-living-ecological space in Shanxi Province, China. Sci. Rep. 14, 31379. doi: 10.1038/s41598-024-82906-6 39733094 PMC11682281

[B13] Muthoni J. Nyamongo D. O. (2008). Seed systems in Kenya and their relationship to on-farm conservation of food crops. J. New Seeds 9, 330–342. doi: 10.1080/1522886080249227337339054

[B14] Navreet Kaur R. Ravika Rajesh Y. Amit Karuna Deepak K. (2024). Morphological characterization and diversity assessment of mungbean [Vigna radiata (L.) Wilczek] genotypes using DUS descriptors as per PPV and FRA, 2001. Legume Research: An. Int. J. 3, 361–9. doi: 10.18805/LR-5264

[B15] Osuman A. S. Badu-Apraku B. Karikari B. Ifie B. E. Tongoona P. Danquah E. Y. . (2022). Genome-wide association study reveals genetic architecture and candidate genes for yield and related characteristic under terminal drought, combined heat and drought in tropical maize germplasm. Genes 13, 349. doi: 10.3390/genes13020349 35205393 PMC8871853

[B16] Palaniyappan S. Ganesan K. N. Manivannan N. Ravichandran V. Senthil N. (2023). Multi char genotype-ideotype distance index - A tool for identification of elite parental inbreds for developing heterotic hybrids of fodder maize (Zea mays L.). Archives 14, 841–849. doi: 10.37992/2023.1403.098

[B17] Pramanik K. Sahu G. S. Acharya G. C. Tripathy P. Dash M. Kumar M. . (2023). DUS characterization and evaluation of untapped French bean (Phaseolus vulgaris L.) genotypes. Int. J. Environ. Clim. Change 13, 225–43. doi: 10.9734/ijecc/2023/v13i11893

[B18] Shen T. Li J. Wang C. Fan H. Zheng Y. Liu Y. . (2025). Evaluation of the kernel test weight and selection of identification indexes of maize inbred lines. Agronomy 15, 1807. doi: 10.3390/agronomy1508180730654563

[B19] Shu G. Wang A. Wang X. Chen R. Gao F. Wang A. . (2023). Identification of QTNs, QTN-by-environment interactions for plant height and ear height in maize multi-environment GWAS. Front. Plant Sci. 14, 1284403. doi: 10.3389/fpls.2023.1284403 38093996 PMC10716222

[B20] Silveira D. L. Filho A. C. de Souza J. M. Trivisiol V. S. Somavilla F. M. (2024). Adaptability and stability of grain yield and maize tassel characteristic. Ciec. Rural 54, e20230395. doi: 10.1590/0103-8478cr2023039541099703

[B21] Song L. Liu G. Yang Y. Guo X. Zhang H. Lu T. . (2024). Drip fertigation improves maize yield, resource utilization, and economic benefits by increasing light interception under dense planting in Southwest China. Food Energy Secur. 13, e70022. doi: 10.1002/fes3.7002242587375

[B22] Su J. Rui H. Zhu Q. Zhang W. Wu Q. Li S. . (2023). Temporal and spatial evolution of habitat quality and its topographic gradient effect in Xinzhou City based on InVEST model from 1985 to 2020. J. Ecol. Rural Environ. 39, 1525–1533. doi: 10.19741/j.issn.1673-4831.2023.0530

[B23] Tibbs-Cortes L. E. Guo T. Andorf C. M. Li X. Yu J. (2024). Comprehensive identification of genomic and environmental determinants of phenotypic plasticity in maize. Genome Res. 34, 1253–63. doi: 10.1101/gr.279027.124 PMC11444181 39271292

[B24] Uddin M. S. Akter F. Azam M. G. Bagum S. A. Hossain N. Billah M. . (2023). Evaluation of inbred maize (Zea mays L.) for tolerance to low phosphorus at the seedling stage. Plants 12, 2520. doi: 10.3390/plants12132520 37447080 PMC10346422

[B25] UPOV (2002). General introduction to the examination of distinctness, uniformity and stability and the development of harmonized descriptions of new varieties of plants (TG/1/3) (Geneva: International Union for the Protection of New Varieties of Plants). Available online at: https://www.upov.int/edocs/tgdocs/en/tg_1_3.pdf.

[B26] Yang F. Liu Z. Wang Y. Wang X. Zhang Q. Zhang Q. . (2023). A variety test platform for the standardization and data quality improvement of crop variety tests. Front. Plant Sci. 14, 1077196. doi: 10.3389/fpls.2023.1077196 36760650 PMC9902355

[B27] Yang C. J. Russell J. Ramsay L. Thomas W. Powell W. Mackay I. . (2021). Overcoming barriers to the registration of new plant varieties under the DUS system. Commun. Biol. 4, 302. doi: 10.1038/s42003-021-01840-9 33686157 PMC7940638

[B28] Yang T. Zhao J. Fu Q. (2024). Quantitative relationship of plant height and leaf area index of spring maize under different water and nitrogen treatments based on effective accumulated temperature. Agronomy 14, 1018. doi: 10.3390/agronomy1405101830654563

[B29] Yao Z. Z. Yang Y. Zi Z. L. . (2025). DUS test for standard varieties of maize grown in ecological regions on low latitude plateaus. Fujian J. Agric. Sci. 40, 45–52. doi: 10.19303/j.issn.1008-0384.2025.01.006

[B30] Yu J. Bai X. Zhang K. Feng L. Yu Z. Jiao X. . (2024). Assessment of breeding potential of foxtail millet varieties using a TOPSIS model constructed based on distinctness, uniformity, and stability test characteristics. Plants 13, 2102. doi: 10.3390/plants13152102 39124220 PMC11314239

[B31] Yue H. Olivoto T. Bu J. Li J. Wei J. Xie J. . (2022). Multi-char selection for mean performance and stability of maize hybrids in mega-environments delineated using envirotyping techniques. Front. Plant Sci. 13, 1030521. doi: 10.5281/zenodo.7221255 36452111 PMC9702090

[B32] Zhang Y. Liu J. Guan L. Fan D. Xia F. Wang A. . (2023). By‐products of Zea mays L.: A promising source of medicinal properties with phytochemistry and pharmacological activities: A comprehensive review. Chem. Biodivers. 20, e202200940. doi: 10.1002/cbdv.202200940 36721262

[B33] Zhang L.-S. Wen H.-Q. Cheng T.-L. Li S.-H. Jia Y.-S. (2010). Ecological regionalization of wheat cultivation in Shanxi Province of China. Chin. J. Eco-Agriculture 18, 410–5. doi: 10.3724/SP.J.1011.2010.00410

[B34] Zhang J. Yang J. Fu S. Ren J. Zhang X. Xia C. . (2022). Comparison of DUS testing and SNP fingerprinting for variety identification in cucumber. Hortic. Plant J. 8, 575–582. doi: 10.1016/j.hpj.2022.07.00242574925

[B35] Zhao J. Yang X. Lv S. Liu Z. Wang J. (2014). Variability of available climate resources and disaster risks for different maturity types of spring maize in Northeast China. Reg. Environ. Change 14, 17–26. doi: 10.1007/s10113-013-0476-930311153

[B36] Zhu T. Feng Y. Dong X. Yang X. Liu B. Yuan P. (2024). Optimizing DUS testing for Chimonanthus praecox using feature selection based on a genetic algorithm. Front. Plant Sci. 14, 1328603. doi: 10.3389/fpls.2023.1328603 38312354 PMC10835806

