# Supplementary material for: Spatiotemporal stability of DUS characteristics in maize and evaluation of spatial replication testing models
Source: Front Plant Sci. 2026 Sep 28;17:1854476. doi: 10.3389/fpls.2026.1854476 (PMC13655061; doi:10.3389/fpls.2026.1854476)
Supplement: Supplementary file 1 [file Supplementaryfile1.docx]

Supplementary Material

# Supplementary Table 1

|  | Material Code | Material Type |  | Material Code | Material Type |  | Material Code | Material Type |
| --- | --- | --- | --- | --- | --- | --- | --- | --- |
| 1 | 20245330001A | Hybrid variety | 41 | 20245330049A | Hybrid variety | 81 | 20245330427A | Hybrid variety,Sweet Corn |
| 2 | 20245330002A | Hybrid variety | 42 | 20245330050A | Hybrid variety | 82 | 20245330428A | Hybrid variety,Sweet Corn |
| 3 | 20245330003A | Hybrid variety | 43 | 20245330051A | Hybrid variety | 83 | 20245330032A | Hybrid variety |
| 4 | 20245330004A | Hybrid variety | 44 | 20245330053A | Hybrid variety | 84 | 20245330033A | Hybrid variety |
| 5 | 20245330005A | Hybrid variety | 45 | 20245330054A | Hybrid variety | 85 | 20245330035A | Hybrid variety |
| 6 | 20245330006A | Hybrid variety | 46 | 20245330055A | Hybrid variety | 86 | 20245330052A | Hybrid variety |
| 7 | 20245330007A | Hybrid variety | 47 | 20245330056A | Hybrid variety | 87 | 20245330060A | Hybrid variety |
| 8 | 20245330009A | Hybrid variety | 48 | 20245330057A | Hybrid variety | 88 | 20245330103A | Hybrid variety,Sweet Corn |
| 9 | 20245330010A | Hybrid variety | 49 | 20245330058A | Hybrid variety | 89 | 20245330437A | Hybrid variety,Waxy Corn |
| 10 | 20245330011A | Hybrid variety | 50 | 20245330059A | Hybrid variety | 90 | 20245330421A | Hybrid variety,Sweet-Waxy Corn |
| 11 | 20245330012A | Hybrid variety | 51 | 20245330061A | Hybrid variety | 91 | 20225330081A | Hybrid variety |
| 12 | 20245330013A | Hybrid variety | 52 | 20245330062A | Hybrid variety | 92 | 20225330081B | Hybrid variety |
| 13 | 20245330014A | Hybrid variety | 53 | 20245330063A | Hybrid variety | 93 | 20235330034A | Hybrid variety,Waxy Corn |
| 14 | 20245330015A | Hybrid variety | 54 | 20245330064A | Hybrid variety | 94 | 20235330034B | Hybrid variety,Waxy Corn |
| 15 | 20245330016A | Hybrid variety | 55 | 20245330065A | Hybrid variety | 95 | 20235330025A | Hybrid variety,Waxy Corn |
| 16 | 20245330017A | Hybrid variety | 56 | 20245330066A | Hybrid variety | 96 | 20235330025B | Hybrid variety,Waxy Corn |
| 17 | 20245330018A | Hybrid variety | 57 | 20245330067A | Hybrid variety | 97 | 20245330489A | Inbred variety,Waxy Corn |
| 18 | 20245330019A | Hybrid variety | 58 | 20245330423A | Hybrid variety,Sweet Corn | 98 | 20245330490A | Inbred variety,Waxy Corn |
| 19 | 20245330020A | Hybrid variety | 59 | 20245330424A | Hybrid variety,Sweet Corn | 99 | 20245330487A | Inbred variety,Waxy Corn |
| 20 | 20245330021A | Hybrid variety | 60 | 20245330422A | Hybrid variety,Sweet-Waxy Corn | 100 | 20245330491A | Inbred variety,Waxy Corn |
| 21 | 20245330023A | Hybrid variety | 61 | 20245330432A | Hybrid variety,Sweet-Waxy Corn | 101 | 20211004867A | Inbred variety |
| 22 | 20245330024A | Hybrid variety | 62 | 20245330102A | Hybrid variety,Waxy Corn | 102 | 20211004867B | Inbred variety |
| 23 | 20245330026A | Hybrid variety | 63 | 20245330429A | Hybrid variety,Waxy Corn | 103 | 20211002612A | Inbred variety |
| 24 | 20245330028A | Hybrid variety | 64 | 20245330430A | Hybrid variety,Waxy Corn | 104 | 20211002612B | Inbred variety |
| 25 | 20245330030A | Hybrid variety | 65 | 20245330431A | Hybrid variety,Waxy Corn | 105 | 20232002438A | Inbred variety |
| 26 | 20245330031A | Hybrid variety | 66 | 20245330433A | Hybrid variety,Waxy Corn | 106 | 20221003104A | Inbred variety |
| 27 | 20245330034A | Hybrid variety | 67 | 20245330434A | Hybrid variety,Waxy Corn | 107 | 20221003104B | Inbred variety |
| 28 | 20245330036A | Hybrid variety | 68 | 20245330435A | Hybrid variety,Waxy Corn | 108 | 20221003104C | Inbred variety |
| 29 | 20245330037A | Hybrid variety | 69 | 20245330438A | Hybrid variety,Waxy Corn | 109 | 2021101338A | Inbred variety |
| 30 | 20245330038A | Hybrid variety | 70 | 20245330439A | Hybrid variety,Waxy Corn | 110 | 2021101338B | Inbred variety |
| 31 | 20245330039A | Hybrid variety | 71 | 20245330099A | Hybrid variety,Sweet Corn | 111 | 20235330004A | Inbred variety |
| 32 | 20245330040A | Hybrid variety | 72 | 20245330426A | Hybrid variety,Sweet Corn | 112 | 20225330003A | Inbred variety |
| 33 | 20245330041A | Hybrid variety | 73 | 20245330425A | Hybrid variety,Sweet Corn | 113 | 20225330003B | Inbred variety |
| 34 | 20245330042A | Hybrid variety | 74 | 20245330418A | Hybrid variety,Sweet-Waxy Corn | 114 | 20211001761A | Inbred variety |
| 35 | 20245330043A | Hybrid variety | 75 | 20245330440A | Hybrid variety,Waxy Corn | 115 | 20211001761B | Inbred variety |
| 36 | 20245330044A | Hybrid variety | 76 | 20245330419A | Hybrid variety,Sweet Corn | 116 | 20225330008A | Inbred variety |
| 37 | 20245330045A | Hybrid variety | 77 | 20245330436A | Hybrid variety,Waxy Corn |  |  |  |
| 38 | 20245330046A | Hybrid variety | 78 | 20245330345A | Hybrid variety,Waxy Corn |  |  |  |
| 39 | 20245330047A | Hybrid variety | 79 | 20245330101A | Hybrid variety,Waxy Corn |  |  |  |
| 40 | 20245330048A | Hybrid variety | 80 | 20245330420A | Hybrid variety,Waxy Corn |  |  |  |

## Supplementary Figures

Frequency distribution of 12 quantitative traits in 116 maize accessions evaluated at Datong in 2025.

Frequency distribution of 12 quantitative traits in 116 maize accessions evaluated at Xinzhou in 2025.

Frequency distribution of 12 quantitative traits in 116 maize accessions evaluated at Xinzhou in 2024.
